# Treadmill Exercise Induces Hippocampal Astroglial Alterations in Rats

**DOI:** 10.1155/2013/709732

**Published:** 2013-01-17

**Authors:** Caren Bernardi, Ana Carolina Tramontina, Patrícia Nardin, Regina Biasibetti, Ana Paula Costa, Adriana Fernanda Vizueti, Cristiane Batassini, Lucas Silva Tortorelli, Krista Minéia Wartchow, Márcio Ferreira Dutra, Larissa Bobermin, Patrícia Sesterheim, André Quincozes-Santos, Jaqueline de Souza, Carlos Alberto Gonçalves

**Affiliations:** ^1^Programa de Pós-Graduação em Neurociências, Instituto de Ciências Básicas da Saúde, Universidade Federal do Rio Grande do Sul, 90046-900 Porto Alegre, RS, Brazil; ^2^Departamento de Bioquímica, Instituto de Ciências Básicas da Saúde, Universidade Federal do Rio Grande do Sul, 90035-003 Porto Alegre, RS, Brazil

## Abstract

Physical exercise effects on brain health and cognitive performance have been described. Synaptic remodeling in hippocampus induced by physical exercise has been described in animal models, but the underlying mechanisms remain poorly understood. Changes in astrocytes, the glial cells involved in synaptic remodeling, need more characterization. We investigated the effect of moderate treadmill exercise (20 min/day) for 4 weeks on some parameters of astrocytic activity in rat hippocampal slices, namely, glial fibrillary acidic protein (GFAP), glutamate uptake and glutamine synthetase (GS) activities, glutathione content, and S100B protein content and secretion, as well as brain-derived neurotrophic factor (BDNF) levels and glucose uptake activity in this tissue. Results show that moderate treadmill exercise was able to induce a decrease in GFAP content (evaluated by ELISA and immunohistochemistry) and an increase in GS activity. These changes could be mediated by corticosterone, whose levels were elevated in serum. BDNF, another putative mediator, was not altered in hippocampal tissue. Moreover, treadmill exercise caused a decrease in NO content. Our data indicate specific changes in astrocyte markers induced by physical exercise, the importance of studying astrocytes for understanding brain plasticity, as well as reinforce the relevance of physical exercise as a neuroprotective strategy.

## 1. Introduction

Studies have shown that physical exercise can have profound effects on cardiovascular, pulmonary, and the musculoskeletal system, as well as the central nervous system (CNS) [1, 2]. Moderate physical activity improves memory and learning [3–8] and is associated with a lower risk for Alzheimer's dementia [9], Parkinson's disease [10] and other types of neurodegenerative diseases [2]. One of the regions of CNS more affected by exercise is the hippocampus. A putative mechanism, through which the exercise exerts its effects on the hippocampus, is the induction of brain-derived neurotrophic factor (BDNF) [11].

Astrocytes, the most abundant glial cells, particularly those of the glutamatergic type, are very important elements in neurotransmission [12, 13] and antioxidant defense, and this action involves the synthesis and secretion of glutathione (GSH) [14]. These cells are responsible for glutamate removal from the synaptic cleft and its conversion, through glutamine synthetase (GS) catalysis, into glutamine for replacement in the neurons. Moreover, increments in energy demand and functional activity during exercise may require functional and structural alterations in astrocytes [15, 16], due to their involvement in the blood brain barrier [17].

The involvement of astrocytes in brain plasticity is commonly related to changes in specific proteins, such as glial fibrillary acidic protein (GFAP), S100B, glutamate transporters and glutamine synthetase. With regard to BDNF, it is important to mention that this neurotrophin, which is *de novo* synthesized in neurons, is released as mature BDNF and pro-BDNF, and that pro-BDNF is uptaken and released by astrocytes [18]. Therefore, recycling of BDNF by astrocytes seems to contribute to the regulation of synaptic plasticity [19].

Physical exercise induces biochemical alterations in astrocytes [20–23] and enhances the behavioral performance of animals in spatial memory tasks [7, 20, 24]. The contribution of astrocytes to the brain plasticity induced by physical exercise needs further characterization. This study aimed to investigate the effect of moderate exercise for 4 weeks on some parameters of astrocytic activity in the rat hippocampus.

## 2. Material and Methods

### 2.1. Animals

Forty-eight male Wistar rats (90 days old, weighing 250–320 g) were obtained from our breeding colony (at the Department of Biochemistry, Federal University of Rio Grande do Sul) and were maintained under controlled light and environmental conditions (12 h light/12 h dark cycle at a constant temperature of 22 ± 1°C) with free access to food and water. All animal experiments were carried out in accordance with the Directive 2010/63/EU of the European parliament and following the regulations of the local animal house authorities. Rats were habituated with the treadmill apparatus to minimize novelty stress and submitted to maximal exercise test. After the test, rats that refused to run or who had the worst performance were assigned to the control/sedentary group (SED) (*N* = 24). The other animals were assigned to the exercise group (EXE) (*N* = 24). Three sets of experiments were carried out separately (Table 1).

### 2.2. Adaptation to Treadmill and Maximal Exercise Test

To perform the training, all animals were adapted to walk on a treadmill for three consecutive days (days 1 to 3). This adaptation consisted of walking on a treadmill for 10 min at 5 m/min. On the fourth day, animals were submitted to the maximal exercise test (MET). The MET was used to determine the maximal exercise capacity (MEC). The test consisted of a graded exercise on the treadmill, with speed increments of 5 m/min every 3 min, starting at 5 m/min and continuing up to the MEC of each rat [25]. Values attained in the MET were used to plan the treadmill training program. 

### 2.3. Treadmill Training

The exercise training consisted of running sessions on an adapted motorized rodent treadmill (INBRAMED TK 01, Porto Alegre, Brazil) at 60% of MEC (maximal velocity). Treadmill training was performed between 8:00 and 12:00 h. The animals ran for 20 minutes, 5 days a week, for 4 weeks. Selected animals that initially refused to run were encouraged by gently tapping their backs. Neither electric shock nor physical prodding was used in this study. The control/sedentary group was transported to the experimental room and handled exactly as the experimental animals were and maintained in the turned off treadmill for 5 min without forcing them to run [26].

### 2.4. Serum Samples

Two hours after the last exercise session (20 min/day, 5 days/week during four weeks) animals (*n* = 5-6) were anesthetized with ketamine and xylazine (75 and 10 mg/kg, resp., i.p.), and then whole blood was obtained through an intracardiac puncture using a 0.37 mm diameter needle that was inserted into the intercostal space above the sternum. Serum was separated by centrifugation at 3000 ×g for 5 min. Serum samples were frozen (−20°C) until further analysis [27].

### 2.5. Hippocampal Tissue Samples

Twenty-four hours after the last exercise session (20 min/day, 5 days/week during four weeks) the animals were killed by decapitation, brains were removed and placed in cold saline medium with the following composition (in mM): 120 NaCl; 2 KCl; 1 CaCl_2_; 1 MgSO_4_; 25 HEPES; 1 KH_2_PO_4_ and 10 glucose, adjusted to pH 7.4, and previously aerated with O_2_. The hippocampi were dissected out, and transverse slices of 0.3 mm were obtained using a McIlwain Tissue Chopper. Slices were transferred immediately to 24-well culture plates, each well contains 0.3 mL of the solution described previously for measuring S100B secretion and 0.3 mL of HBSS (Hank's balanced salt solution) for measuring glutamate uptake and glucose uptake. Slice samples (hippocampus) were then frozen (−80°C) for biochemical measurements, described as follows.

### 2.6. Quantification of S100B and GFAP

S100B content in the hippocampus was measured by ELISA [28]. Briefly, 50 *μ*L of sample plus 50 *μ*L of tris buffer were incubated for 2 h on a microtiter plate previously coated with monoclonal anti-S100B (SH-B1). Polyclonal anti-S100B was incubated for 30 min and then peroxidase-conjugated anti-rabbit antibody was added for a further 30 min. A colorimetric reaction with o-phenylenediamine was measured at 492 nm. The standard S100B curve ranged from 0.019 to 10 ng/mL. ELISA for GFAP [29] was carried out by coating the microtiter plate with 100 *μ*L samples containing 30 *μ*g of protein for overnight at 4°C. Incubation with a rabbit polyclonal anti-GFAP for 2 h was followed by incubation with a secondary antibody conjugated with peroxidase for 1 h, at room temperature; the standard GFAP curve ranged from 0.1 to 10 ng/mL.

### 2.7. Immunohistochemistry for GFAP

Rats were anesthetized using ketamine/xylazine (75 and 10 mg/kg, resp., i.p.) and were perfused through the left cardiac ventricle with 200 mL of saline solution, followed by 200 mL of 4% paraformaldehyde in 0.1 M phosphate buffer saline (PBS), pH 7.4. After perfusion, the brains were removed, postfixed in the same fixative solution for 4 h at room temperature, and cryoprotected by immersion in 15% and 30% sucrose solution in PBS at 4°C. The brains were then frozen by immersion in isopentane cooled with CO_2_ and stored in a freezer (−80°C) for later analyses [30]. Coronal slices (45 *μ*m) were obtained at −20°C using a cryostat (Leitz). The free-floating sections were incubated with polyclonal anti-GFAP from rabbit, diluted 1 : 3000 in PBS-Triton X-100 0.4% and 2% bovine serum albumin (BSA), for 48 h at 4°C. After washing several times in PBS, tissue sections were incubated with anti-rabbit Alexa 488 (Molecular Probes) diluted 1 : 500 in PBS-TX 0.1% and 2% BSA for 1 h at room temperature. Afterwards, the sections were washed several times in PBS, mounted on slides with Fluor Save, and covered with coverslips. The images were obtained with a Confocal Olympus IX-81microscope.

### 2.8. Glutamate Uptake Assay

Glutamate uptake was performed as previously described [31]. Briefly, hippocampal slices were transferred to 24-well plates and incubated for 23 min at 37°C in a Hank's balanced salt solution (HBSS) containing (in mM): 137 NaCl, 5.36 KCl, 1.26 CaCl_2_, 0.41 MgSO_4_, 0.49 MgCl_2_, 0.63 Na_2_HPO_4_·7H_2_O, 0.44 KH_2_PO_4_, 4.17 NaHCO_3_, and 5.6 glucose, adjusted to pH 7.4. The assay was started by the addition of 0.1 mM L-glutamate and 0.33 *μ*Ci/mL L-[2, 3-^3^H] glutamate. Incubation was stopped after 5 min by removal of the medium and rinsing the slices twice with ice-cold HBSS. The slices were then lysed in a solution containing 0.5 M NaOH. Radioactivity was measured in a scintillation counter. Sodium-independent uptake was determined using N-methyl-D-glucamine instead of NaCl. Sodium-dependent glutamate uptake was obtained by subtracting the nonspecific uptake of the total uptake to obtain the specific uptake. Results (nmol/mg protein/min) were expressed as a percentage of the control.

### 2.9. Glutamine Synthetase (GS) Activity

The enzymatic assay was performed, as described previously [32]. Briefly, homogenized tissue samples were added to a reaction mixture containing (in mM): 10 MgCl_2_; 50 L-glutamate; 100 imidazole-HCl buffer (pH 7.4); 10 2-mercaptoethanol; 50 hydroxylamine-HCl; 10 ATP and incubated for 15 min at 37°C. The reaction was stopped by the addition of 0.4 mL of a solution containing: 370 mM ferric chloride; 670 mM HCl; 200 mM trichloroacetic acid. After centrifugation, the supernatant was measured at 530 nm and compared to the absorbance generated by standard quantities of *γ*-glutamylhydroxamate treated with ferric chloride reagent. Glutamine synthetase activity was expressed as *μ*mol/mg prot/min.

### 2.10. Glutathione (GSH) Content Assay

GSH levels were measured as previously described [33]. This assay detects only the reduced glutathione content. Briefly, homogenized slices were assayed in sodium phosphate buffer (0.1 M, pH 8.0), and protein was precipitated with 1.7% meta-phosphoric acid. Supernatant was assayed with *o*-phthaldialdehyde (1 mg/mL methanol) at room temperature for 15 min. Fluorescence was measured using excitation and emission wave lengths of 350 and 420 nm, respectively. A calibration curve was performed with standard glutathione solutions (0–500 *μ*M). Glutathione concentrations were expressed as nmol/mg protein.

### 2.11. NO Assay

NO metabolites, NO_3_-(nitrate), and NO_2_-(nitrite) were determined according to [34]. Briefly, homogenates from hippocampal were mixed with 25% trichloroacetic and centrifuged at 1800 g for 10 min. The supernatant was immediately neutralized with 2 M potassium bicarbonate. NO_3_^−^ was reduced to NO_2_^−^ by nitrate reductase. The total NO_2_^−^ in the incubation was measured by a colorimetric assay at 540 nm, based on the Griess reaction. A standard curve was performed using sodium nitrite (0–80 *μ*M). Results were expressed as *μ*M of nitrite/mg protein.

### 2.12. Evaluation of Intracellular Reactive Oxygen Species (ROS) Production

Intracellular ROS production was detected using the nonfluorescent cell permeating compound, 2′–7′-dichlorofluorescein diacetate (DCF-DA). Samples homogenized in sodium phosphate buffer, pH 7.4 with 140 mM KCl were treated with DCF-DA (10 *μ*M) for 30 min at 37°C. The fluorescence was measured in a plate reader (Spectra Max GEMINI XPS, Molecular Devices, USA) with excitation at 485 nm and emission at 520 nm, as described previously [35]. Values are obtained as a unit of fluorescence/mg protein and are expressed as percentage of control.

### 2.13. Glucose Uptake

Glucose uptake was performed in hippocampal slices. Briefly, slices were transferred to 24-well plates and incubated for 30 min at 37°C in a Hank's balanced salt solution (HBSS) containing (in mM): 137 NaCl, 5.36 KCl, 1.26 CaCl_2_, 0.41 MgSO_4_, 0.49 MgCl_2_, 0.63 Na_2_HPO_4_·7H_2_O, 0.44 KH_2_PO_4_, 4.17 NaHCO_3_, and 5.6 glucose, adjusted to pH 7.4. The assay was started by addition of 0.1 *μ*Ci/mL [2,3-^3^H]-D-glucose. The incubation was stopped after 30 min by removal of the medium and rinsing the slices twice with ice-cold HBSS. The slices were then lysed in a solution containing 0.5 M NaOH. Radioactivity was measured in a scintillation counter. Nonspecific uptake was determined by using 25 *μ*M cytochalasin B. Final glucose uptake was obtained by subtracting the nonspecific uptake of the total one to obtain the specific uptake.

### 2.14. Corticosterone Enzyme Immunoassay (EIA)

Corticosterone concentrations in serum were measured using a commercial EIA kit conforming the manufacturer's protocol (Cayman Chemicals, MI, USA). Briefly, 50 *μ*L aliquots were added to 96-well plates precoated with rabbit antisheep IgG antibody. We added corticosterone-specific acetylcholinesterase tracer and specific corticosterone antiserum and then placed plates on an orbital shaker for two hours. Plates were then developed and read at 412 nm. The concentration of corticosterone was calculated by comparing samples to the standard curve generated with the kit. 

### 2.15. BDNF Concentration

BDNF protein was assessed using the ChemiKine brain-derived neurotrophic factor (BDNF) Sandwich ELISA kit (Millipore, USA), according to the manufacturer's recommendations. Briefly, the slices of hippocampus were individually homogenized in buffer containing 100 mM Tris-HCl (pH 7.0), containing 2% bovine serum albumin (BSA), 1 M NaCl, 4 mM EDTA·Na_2_, 2% Triton X-100, 0.1% sodium azide, and the cocktail protease inhibitors (Sigma). Samples were centrifuged for 30 min at 14,000 ×g. The supernatant was incubated on a 96-well microplate previously coated with anti-BDNF monoclonal antibody. After blocking, plates were incubated with biotinylated mouse anti-BDNF monoclonal antibody for 2 h and streptavidin-HRP conjugate solution for 1 h. Then color reaction with 3,3′,5,5′-tetramethylbenzidine substrate was quantified in a plate reader at 450 nm. The standard BDNF curve ranged from 7.8 to 500 pg/mL.

### 2.16. Lactate Measurement

An increase in lactate level (reflecting an improved muscle metabolism) in the blood is commonly used as a marker of the effect of exercise training [36]. Blood samples were collected from a cut at the tip of the tail at the end of the last exercise bout. The lactate concentrations were determined with a lactate analyzer (*Accutrend Plus*, Roche Diagnostic, Germany).

### 2.17. Statistical Analysis

Parametric data from the experiments are presented as means ± standard error and statistically evaluated by Student's *t*-test, assuming *P* < 0.05.

## 3. Results

### 3.1. Exercise Protocol Characterization

**Figure 1:**
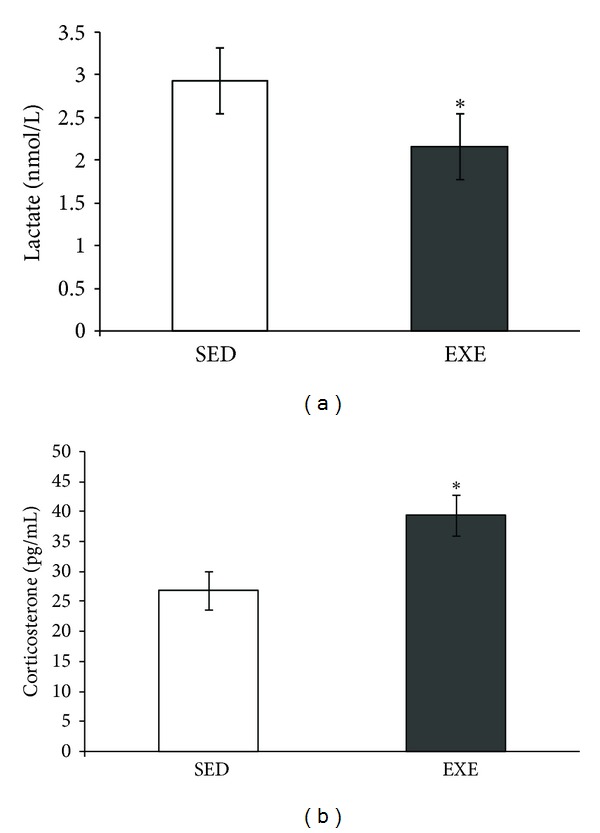
Moderate treadmill exercise increased serum corticosterone but decreased lactate levels. The blood lactate and serum corticosterone levels of rats submitted to 4 weeks (20 min/day, 5 days/week during four weeks) of treadmill exercise. The blood lactate level was measured with a lactate analyzer (a), and serum corticosterone was measured by ELISA (b). Values are mean ± standard error of control/sedentary (SED) (*n* = 5) and exercise (EXE) group (*n* = 6). *means significantly different from other group (Student's *t*-test, *P* < 0.05).

In order to characterize whether the protocol for the treadmill exercise was aerobic, we analyzed the blood lactate levels in animals [37]. The concentration of blood lactate in both groups, exercise (EXE) and control/sedentary (SED), remained below the anaerobic threshold of 7.17 ± 0.16 mM. Moreover, a significant decrease in blood lactate levels was observed in the exercise group, as compared to the control/sedentary group (*P* = 0.04) (Figure 1(a)). Serum corticosterone was used to evaluate stress responses induced by this exercise protocol. A small but significant increase was observed in serum corticosterone levels (*P* = 0.04, Figure 1(b)).

### 3.2. Hippocampal Contents of BDNF and GFAP

**Figure 2:**
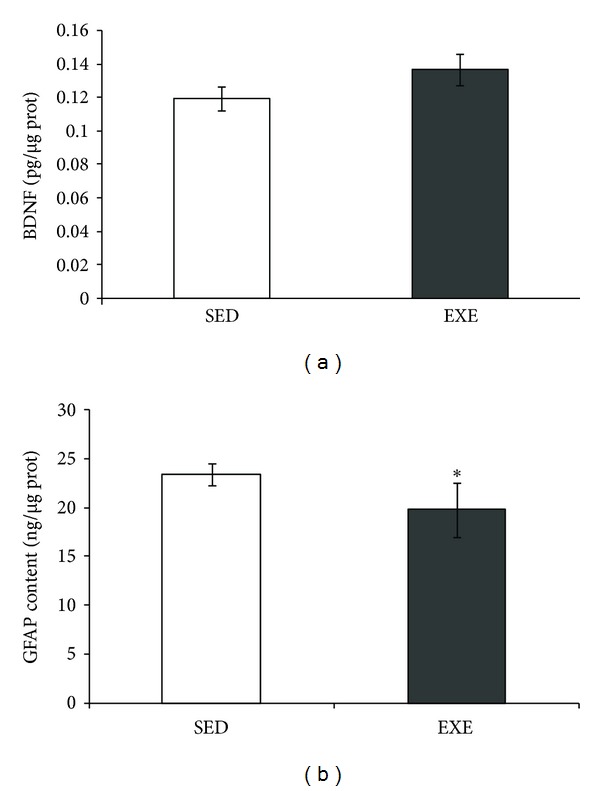
Moderate treadmill exercise decreased GFAP content in the hippocampus of exercised rats. Effects of treadmill exercise (20 min/day, 5 days/week during four weeks) on BDNF (a) and GFAP (b) contents in rat hippocampus were evaluated and were measured by ELISA. Values are mean ± standard error of control/sedentary (SED) (*n* = 6) and exercise (EXE) group (*n* = 7). *means significantly different from respective control/sedentary group (Student's *t*-test, *P* < 0.05).

**Figure 3:**
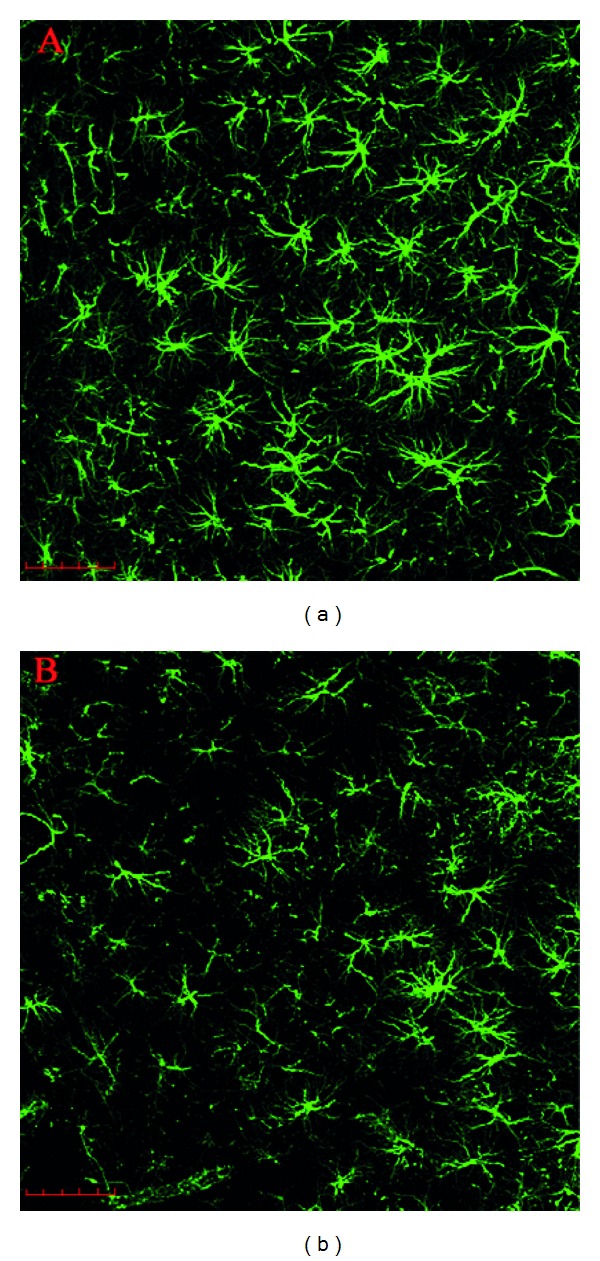
Immunohistochemistry for GFAP from rats submitted to treadmill exercise. Serial stack images obtained with an Olympus Confocal FV-1000 from GFAP immunofluorescence staining in the *stratum radiatum* of CA1 hippocampal region of rats submitted to treadmill exercise (20 min/day, 5 days/week during four weeks). The images showed an evident reduction in GFAP expression in control/sedentary (SED) rats (a) when compared to exercised (EXE) rats (b). Magnification of 40x, 1.0 *μ*m of optical stack thickness, and 15 confocal planes. Scale bars = 50 *μ*m.

The BDNF content in hippocampus was not changed by this exercise protocol (*P* = 0.17, Figure 2(a)). However, the mean GFAP content was decreased in exercised rats (*P* = 0.02, Figure 2(b)). The immunohistochemistry of the *stratum radiatum* of CA1 hippocampal region (Figure 3(a) and 3(b)) suggests a decrease in GFAP immunoreactivity.

### 3.3. S100B Content and Secretion in Hippocampal Slices

**Figure 4:**
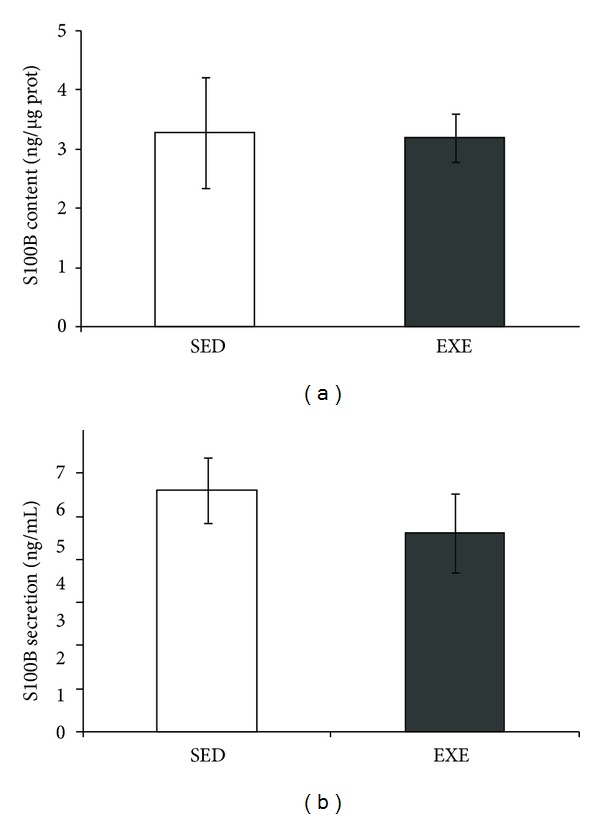
Hippocampal content and secretion of S100B were not affected by moderate treadmill training. S100B content and secretion in hippocampal slices of rats submitted to moderate treadmill exercise (20 min/day, 5 days/week during four weeks) were analyzed. Hippocampi were dissected out and chopped into 0.3 mm slices for measurement of total S100B content (a) and basal secretion in 1 h (b). S100B content was measured by ELISA. Values are mean ± standard error of control/sedentary (SED) (*n* = 6) and exercised (EXE) (*n* = 7) rats.

No significant differences in S100B content, as evaluated by ELISA, were observed in the hippocampus of rats submitted to treadmill exercise (*P* = 0.92, Figure 4(a)). Hippocampal slice preparations from the two groups were used to evaluate *in vitro* S100B secretion. No significant changes in basal S100B secretion were found at 1 h (Figure 4(b)).

### 3.4. Glutamate Metabolic Parameters

**Figure 5:**
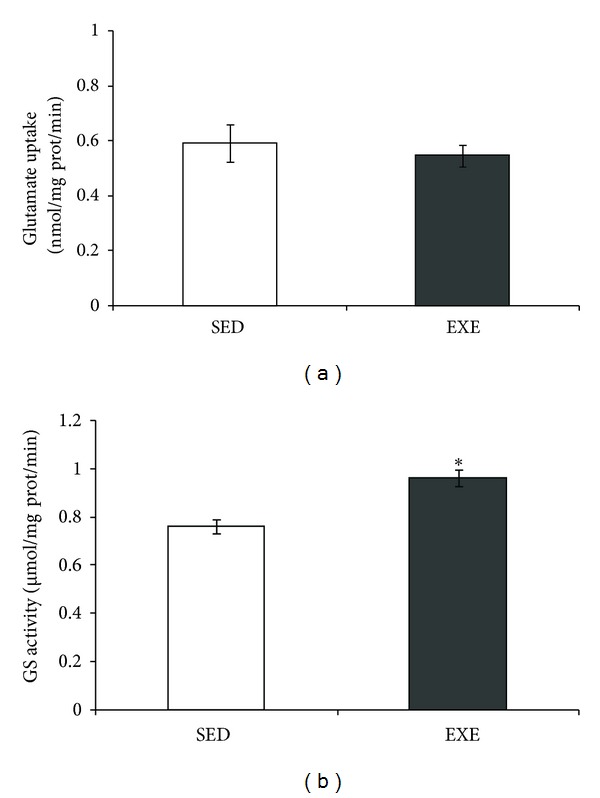
Moderate treadmill exercise increased glutamine synthetase activity in hippocampus of rats. Glutamate uptake and glutamine synthetase (GS) activity in the hippocampus of rats submitted to moderate treadmill exercise (20 min/day, 5 days/week during four weeks) were analyzed. Hippocampi were dissected out and chopped into 0.3 mm slices for measurement of glutamate uptake (a) or homogenized for measurement of GS activity (b). Values are mean ± standard error of control/sedentary (SED) (*n* = 8) and exercise (EXE) (*n* = 9) group. *means significant differences from control (Student's *t*-test, *P* < 0.05).

This exercise protocol did not change the glutamate uptake (*P* = 0.56, Figure 5(a)) in hippocampal tissue. In contrast, we observed an increase in GS activity in exercised rats (*P* = 0.003, Figure 5(b)).

### 3.5. Hippocampal Redox Parameters

**Figure 6:**
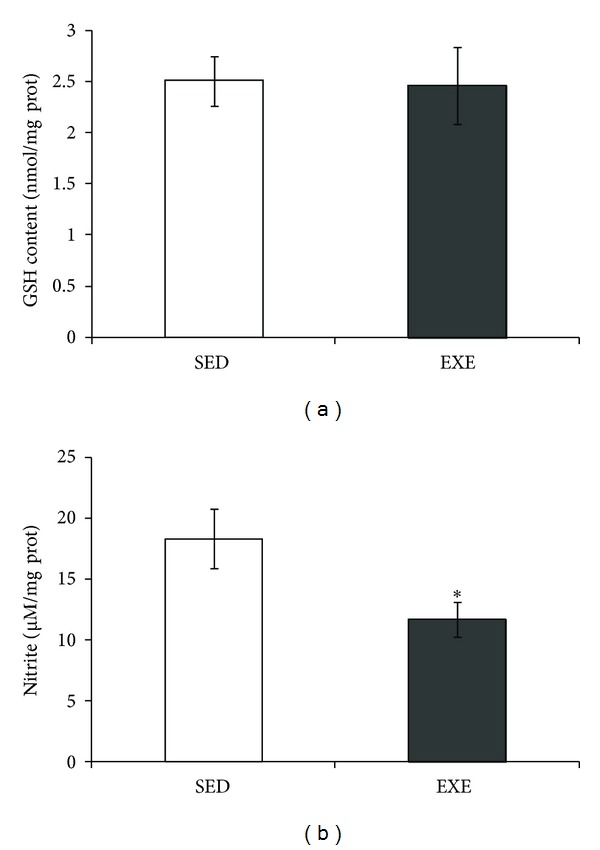
Moderate treadmill exercise decreased NO levels in hippocampus of rats. GSH (reduced form of gluthatione) and NO content in the hippocampus of rats submitted to treadmill exercise (20 min/day, 5 days/week during four weeks) were analyzed. Twenty-four hours after the last session of exercise, hippocampi were dissected out and homogenized for measurement of GSH (a) or NO (b). Values are mean ± standard error of control/sedentary (SED) (*n* = 6) and exercise (EXE) (*n* = 7) group. *means significantly different from respective control/sedentary group (Student's *t*-test, *P* < 0.05).

Two parameters were investigated to evaluate oxidative stress in the hippocampus: GSH (reduced form of glutathione) and NO (nitric oxide) contents. No significant differences were observed in GSH content (*P* = 0.92, Figure 6(a)). Conversely, a decrease in NO content (based on nitrite measurement) was found in the hippocampal tissue of exercised rats (*P* = 0.049, Figure 6(b)).

## 4. Discussion

Physical exercise has unquestionable beneficial effects on brain health. However, relatively little is known about mechanisms underlying these benefits [2, 38]. Synaptic remodeling in hippocampus induced by physical exercise has been described in animal models (e.g., [39]). Astrocytes have been shown to play an important role in synaptic remodeling [40–42] and protecting the CNS against various pathologies [43]. The aim of this study was to evaluate the effect of moderate treadmill running on astroglial parameters in the rat hippocampus. 

Our results show that 4 weeks of moderate exercise decrease the GFAP content in the rat hippocampus. This is in agreement with a decrease in the number and immunoreactivity of GFAP cells in the rat cerebral cortex after running [22]. However, there are some controversies in the literature regarding the effect of physical exercise on GFAP expression in astrocytes. Some studies have shown that physical exercise seems to increase GFAP expression in different brain regions, such as the cerebral cortex, striatum [15], and hippocampus [20]. On the other hand, some studies have not found alterations in this parameter in the rat hippocampus [21, 23]. The discrepancy in results probably arises from methodological differences, including brain regions, exercise protocol (frequency, intensity), and duration of exercise training [44]. Moreover, we cannot rule out that the hippocampal changes observed (on GFAP and other parameters) reflect differences in rats, based on inherent differences; that is, rats that eagerly exercised versus rats that refused to run during the initial exercise test. However, blood lactate levels measured previously to this test were not altered (data not shown). 

Lactate is an important energetic substrate for the brain, and it has been proposed that an increment of blood lactate, during physical activity, could induce brain changes [45]. Interestingly, we found a decrease in serum lactate with our protocol of physical activity. This apparent paradoxical decrease has also been described in mice submitted to 6 weeks of moderate-intensity treadmill exercise [46]. This decrease could be interpreted as an adaptation to exercise, in particular with an improvement of the Cori cycle (between the muscle and liver). In support of this idea, authors found an increase in lactate transporter in the liver, during this study. Therefore, it appears that lactate is not mediating the observed hippocampal changes. However, we cannot exclude an increment of blood lactate at the beginning of the exercise protocol, leading to hippocampal changes.

GFAP is a highly regulated protein, whose expression is induced by brain development and injury [47]. We observed a decrease in hippocampal GFAP, induced by physical exercise. At this moment, our work hypothesis is that corticosterone, which we found increased in our protocol of physical exercise, can downregulate GFAP expression. In fact, GFAP levels are negatively regulated by glucocorticoids in the rat hippocampus (see [48], for a review). Moreover, we can speculate that this downregulation of GFAP (induced by physical exercise) could be associated with other neuronal changes that accompany GFAP downregulated conditions, such as neurite outgrowth [49, 50]. Therefore, we may suggest that GFAP decreases as in indirect signal of neuronal plasticity in the rat hippocampus in response to treadmill exercise. It has been suggested that GFAP expression has an important role in astrocyte-neural interactions [51]; however, the consequence of this change in the cognitive process remains unclear. In contrast to GFAP, S100B was not changed by physical exercise in the rat hippocampus, confirming previous reports [21, 23]. This protein is secreted by astrocytes and has a trophic extracellular effect on neuron and glial cells [52, 53]. Moreover, no changes in basal *in vitro* secretion of this protein in the hippocampal slices were observed, in agreement with unaltered levels of cerebrospinal fluid S100B in rats submitted to treadmill exercise [20].

BDNF is a neurotrophin, associated with brain plasticity that is induced by physical exercise [2, 9, 54]. This neurotrophin had no effect on GFAP expression in the retina [55] but was able to reduce GFAP expression in the cerebral cortex after ischemia [56]. On the other hand, BDNF was able to restore hippocampal GFAP levels reduced by chronic unpredictable mild stress in rats [57]. Under these conditions, we did not observe any significant physical exercise-induced changes in BDNF that could be related to decreased GFAP levels. Corticosterone is able to transiently downregulate BDNF mRNA in the hippocampus [58], possibly contributing, in this study, to maintain the levels of this neurotrophin unaltered. Moreover, other studies using moderate acute physical exercise (for 1 h) [59] (or for 2 weeks) [60, 61] did not find changes in hippocampal BDNF either. 

In addition to the decrease in GFAP, we found an increment in GS activity. Whether this effect also depends on corticosterone remains unclear, but it has been reported that expression of GFAP and GS *in vivo* shows opposite responses to corticosterone in the hippocampus [62]. However, there is not enough information about changes in these proteins induced by physical exercise. Recently, we found that GS activity was increased by caloric restriction but not by physical exercise (treadmill running, 20 min sessions, for 12 weeks) [23]. This could suggest that the GS increment induced by the 4-week protocol of treadmill running could be transitory (as well as GFAP decrease). The increased GS activity induced by physical exercise could be beneficial in the brain; this enzyme is involved in the modulation of the turnover of glutamate through the glutamate-glutamine cycle and detoxification of ammonia, and its decrease has been observed in neurodegenerative disorders [63]. The enzyme activity is negatively modulated by nitration on the tyrosine residue, through a NMDA receptor-mediated cGMP-NO signal transduction pathway (see Butterworth [64] for a review). 

Note that while NO works as a physiological mediator, excessive NO release due to the high expression of inducible NO synthase (iNOS) mediates inflammatory/degenerative brain diseases [65]. Herein, we observed a decrease in NO content (measured by nitrite content), which in turn could be connected to the increased GS activity. In agreement with these findings, physical exercise was able to prevent the increase in hippocampal NO induced by intracerebroventricular streptozotocin administration [20]. The mechanism for this is unclear, but it may also be mediated by corticosterone. In support of this hypothesis, swimming exercise induced a decrease in the rat hippocampal iNOS, possibly dependent on corticosterone [66]. Other experimental evidence reinforces the importance of treadmill exercise for decreasing iNOS in murine models of neurodegenerative diseases, such as Parkinson's [67] and Alzheimer's diseases [68].

Considering the close metabolic relationship between glutamine and glutamate, we also investigated glutamate uptake in hippocampal slices of exercised rats. No changes in glutamate uptake were observed, in agreement with our previous study using a longer protocol of physical exercise [23]. Hippocampal GSH content and ROS (data not shown) were not altered by physical exercise under our conditions. A glutathione decrease could suggest oxidative stress, and an increment induced by physical exercise has been interpreted as signal of improvement in antioxidant defense [20, 23]. An elegant study has shown that moderate treadmill running did not change the basal content of glutathione, but it was able to reverse the decrease in glutathione induced by intraperitoneal buthionine-sulfoximine administration [69]. Another important aspect for brain activity, during physical exercise, is the increment cerebral blood flow that is commonly related to energetic substrate uptake [70]. For example, diabetic type 2 patients may benefit from physical exercise, as muscles dramatically increase glucose uptake [71]. However, brain alterations induced by physical alterations have not been evaluated, and potential changes in the hypometabolism that precedes neurodegenerative diseases (see Cunnane, et al. [72]) could be very useful to improve life quality. Herein, no changes were observed in the glucose uptake of hippocampal slices of healthy exercised animals (data not shown). However, this parameter should be investigated in brain disease models, where physical exercise has been proposed as a neuroprotective strategy (e.g., Rodrigues et al. [20]).

## 5. Conclusions

Our data show that moderate treadmill exercise was able to induce a decrease in GFAP content (evaluated by ELISA and immunohistochemistry) and to increase GS activity. These changes could be mediated by corticosterone, whose levels were elevated in serum. BDNF, another putative regulator of the expression of these astroglial proteins, was not altered in hippocampal tissue. Moreover, treadmill exercise caused a decrease in NO content. We did not observe changes in other astroglial parameters such as glutamate uptake, GSH content, and S100B protein. Taken together, these data indicate specific changes in astrocyte markers induced by physical exercise, the importance of astrocytes in brain plasticity, as well as reinforce the relevance of physical exercise as a strategy for neuroprotection.

## Figures and Tables

**Table 1:** Experimental sets of rats submitted to treadmill exercise.

Experimental set	*N*	Biochemical assays	Samples
1	11	Corticosterone	Blood
2	20	Immunohistochemistry for GFAP; ELISA for GFAP, S100B, and BDNF; GSH and NO measurement	Hippocampal tissue
3	17	Glutamate uptake and S100B secretion in slices	Hippocampal tissue

Adult Wistar rats were submitted to 4 weeks (20 min, 5 times a week) of treadmill exercise. At 2 h (set 1) or 24 h (sets 2 and 3) after the last exercise session, rats were anesthetized, killed, and samples were collected for the biochemical assays, as described in Section 2.
